# Assessing Family Physicians’ Knowledge and Attitudes Toward Malnutrition in the Elderly

**DOI:** 10.1093/geroni/igaf122.2439

**Published:** 2025-12-31

**Authors:** Galia SheferHilel, Josefa Kachal, Danit Shahar, Aya Biderman, Shimon Amar

**Affiliations:** Tel-Hai College, Qiryat Shemona, HaZafon, Israel; Ministry of Haelth, Ashdod, HaDarom, Israel; Ben Gurion University of the Negev, Be’er Sheva, HaDarom, Israel; Ben Gurion University of the Negev, Be’er Sheva, HaDarom, Israel; Ben Gurion University of the Negev, Be’er Sheva, HaDarom, Israel

## Abstract

Malnutrition among older populations significantly impacts healthcare, social, and aged-care systems, and often remains unnoticed and untreated in older populations. This study evaluates family physicians’ knowledge and attitudes toward the diagnosis and treatment of malnutrition in this group. An online questionnaire, developed from a literature review, included seven items on knowledge and eight on attitudes concerning malnutrition in older individuals. We also explored the feasibility of including two malnutrition screening questions in regular clinic visits for people aged 70 years and above. Surveys were completed by 126 physicians (35% response rate), with an average age of 47.2±12.6 years; 15.6±12.5 years of practice; 67% females; and 92% board-certified family physicians. Notably, 77.6% agreed that diagnosing malnutrition is important in patients with decreased appetite. A majority demonstrated knowledge of nutritional screening principles (63.5%) and acknowledged that malnutrition could affect obese older individuals (83.2%). There was partial agreement (60%) that normal BMI values in the older adults differ from those in younger populations. Almost complete agreement (91%) was seen for incorporating two nutritional status questions during medical visits with physicians expressing willingness to undergo training in malnutrition identification and screening tools. Despite challenges such as time constraints and limited knowledge, there was support for biannual screening of older patients for malnutrition risk. Based on our findings. We recommend routine malnutrition screening in primary care, followed by diagnosis and referral for appropriate interventions for malnourished patients aged 70 and above.

